# Awareness, want, and adoption of digital health services among older adults in China: a cross-sectional study

**DOI:** 10.3389/fpubh.2026.1820440

**Published:** 2026-04-24

**Authors:** Sitong Wu, Zheng Wan, Lihui Peng, Qifan Zhang, Xiaoyi Wu, Xiaoying Lin

**Affiliations:** 1 The Second School of Clinical Medicine, Cheeloo College of Medicine, Shandong University, Jinan, China; 2 Department of Neurology, The Second Qilu Hospital of Shandong University, Jinan, China; 3 Medical Integration and Practice Center, Cheeloo College of Medicine, Shandong University, Jinan, Shandong, China

**Keywords:** digital health services, eHealth literacy, older adults, matrix analysis, patient activation, structural equation model, technology acceptance model

## Abstract

**Background:**

Population ageing is increasing the demand for chronic disease management, long-term care, and continuous health services, and has become a major public health challenge. This issue is particularly salient in China, where by 2020 adults aged 60 years and older and 65 years and older accounted for 18.70% and 13.50% of the total population, respectively, while digital health services have expanded rapidly. However, older adults do not automatically benefit from digital transformation, and the digital divide remains widespread. Clarifying the key discontinuities in older adults’ progression from awareness to want and actual adoption of digital health services is important for promoting digital health equity.

**Methods:**

This cross-sectional survey used systematic sampling to recruit 451 patients aged 60 years and older with chronic diseases from tertiary hospitals in Jinan, Shandong. Based on a three-stage framework of awareness, want, and adoption, matrix analysis of the awareness–want–adoption gap was used to examine the digital divide across three functional dimensions of digital health services: access to medical information, convenient medical services, and online health management. A structural equation model was further developed to examine the effects of eHealth literacy, patient activation, and health status on digital health service use.

**Results:**

Older adults showed a pattern of relatively high awareness and want but low actual adoption of digital health services. Overall awareness and want rates were 45.60% and 46.27%, respectively, whereas the adoption rate was only 18.27%. Among the three functional dimensions, online health management had the lowest adoption rate (8.87%) and the largest usage gap (75.23%). Structural equation modeling further showed that eHealth literacy was an important determinant of digital health service use and was significantly associated with both behavioral intention and actual adoption.

**Conclusion:**

The main challenge in digital health service use among older adults lies not only in service availability, but also in the marked discontinuity between awareness and actual adoption. Promoting digital health equity for older adults requires interventions targeting barriers in specific functional domains, particularly online health management, while also improving eHealth literacy, accessibility, and usability.

## 1 Introduction

With the continuous trend of global population ageing, the health of older population has become the major focus of global attention (1). Especially in China, the rapid acceleration of population ageing has brought serious challenges to public health and medical services. In China, this trend has evolved into not merely a demographic change, but a major public health and health-system challenge (2–4). Since the late 1970s, China’s ageing process has accelerated rapidly, with the older population increasing at an estimated annual rate of about 3.2% (5). According to the Seventh National Population Census, by 2020 adults aged 60 years and older and 65 years and older accounted for 18.70% and 13.50% of the total population, respectively (6). This demographic shift has been shaped by declining fertility, increasing life expectancy, and the long-term effects of the one-child policy, which together have altered China’s age structure and family support patterns for older adults (4, 7, 8). As a result, demands for chronic disease management, long-term care, and continuous health services are rising, while traditional family-based support is under growing strain (9). Therefore, clarifying the key discontinuities in older adults’ progression from awareness to intention and actual adoption of digital health services is both a timely research priority and a pressing public health issue in China (3, 10–13). There is an urgent need for innovative and diversified medical technology services to improve the quality of life and well-being of older adults (2, 14). Against this background, digital health services are increasingly viewed as an important strategy to improve access, continuity, and efficiency of care, particularly for older adults and people with chronic conditions.

Digital health services provide online medical services with the great potential of artificial intelligence, big data, and other medical resources. They can help address inefficiency, resource shortages, and uneven distribution in the healthcare system, thereby offering more personalized and convenient services, which is especially relevant for chronic disease management among older adults (15). As of December 2021, the number of Chinese netizens has reached 1.032 billion. In addition, 60% of patients in large hospitals that provide digital health services are positive about such services (16). After the outbreak of COVID-19, digital health services have been rapidly popularized around the world (17). In China, digital health services had already shown strong development potential before the pandemic, and COVID-19 further accelerated their widespread application and promotion (18, 19). Importantly, many internet-based medical functions in China, such as online appointment booking, registration, payment, report checking, and online consultation, have gradually been incorporated into routine hospital service processes (16, 20, 21). This suggests that, in China, digital health services are increasingly embedded within institutional healthcare delivery rather than existing solely as optional external technologies. Cross-national evidence also indicates that acceptance of online health services differs across settings; compared with users in Japan and some other East Asian contexts, Chinese users show a distinct acceptance pattern, with social influence and facilitating conditions playing relatively greater roles in shaping intention to use online medical applications (22). Nevertheless, broader service expansion and institutional integration do not automatically translate into equitable benefit (21, 23). Older adults in China may still face substantial barriers in access and actual use, despite increasing exposure to digital interfaces in everyday healthcare encounters (11, 13, 24).

From the perspective of health, older adults face complex and diverse health problems. A survey of more than 107,000 patients in 19 countries conducted by the Organization for Economic Cooperation and Development (OECD) shows that more than 80% of people aged 45 and above suffer from at least one chronic disease, and more than 50% suffer from two or more chronic diseases (25). In addition, with age, the visual, auditory, cognitive, and perceptual abilities of older adults will gradually decline (26). The superposition of these multiple health challenges leads to a growing and continuous demand for medical services, making older adults a key target population for digital health services.

Although digital health services hold substantial value for older adults, there are still significant differences in the acceptance and utilization of different functional categories. Factors such as physical dysfunction, language barriers, economic difficulties, and low education level are increasingly hindering older adults from adopting and using digital health services, highlighting the significant obstacles brought about by the digital divide faced by this group (27, 28). This is reflected in the differences in willingness to accept digital technology, ability to obtain online services, and operating skills (29). Existing studies have often treated digital health use as a single outcome, while paying less attention to where older adults disengage in the progression from awareness to want and actual adoption (10). Therefore, clarifying the key discontinuities across these stages is both a timely research priority and a pressing public health issue in China.

Liang’s research on the adoption behavior of digital health services innovatively divides the process into three stages: awareness, want, and adoption, emphasizing that gaps at the awareness stage may affect subsequent usage behavior (30). This staged perspective provides a basis for more accurate identification of obstacles faced by older population at different decision-making nodes.

As one of the most influential theoretical frameworks in information system research, the Technology Acceptance Model (TAM) originates from the Theory of Reasoned Action (TRA) (31, 32), In recent years, the Technology Acceptance Model has been widely used in the medical field (32). Through the two core concepts of perceived usefulness (PU) and perceived ease of use (PEOU), the model systematically explains the inherent mechanism of individual acceptance and use of information technology, and provides a concise and powerful theoretical basis for understanding the process of technology adoption (33). Attitude is determined by perceived usefulness and perceived ease of use, and serves as a key mediating variable through which these two constructs influence behavioral intention (BI). Behavioral intention, in turn, affects actual use (AU). In summary, individuals are more inclined to adopt technologies that they perceive as useful and easy to operate. This perception will affect their attitude and behavioral intentions, and then determine their actual use.

Based on Liang’s theory that technology adoption is divided into three stages: awareness, want, and adoption, this study adopts this three-stage framework to conceptualize digital health service utilization in a more fine-grained way. This division helps to identify specific obstacles at various stages of digital health service adoption.

eHealth literacy refers to the ability of individuals to search for, understand and evaluate health information from electronic media and use this knowledge to solve health problems (34). Improving eHealth literacy can promote the acceptance and adoption of digital health services.

Patient activation level is a behavioral concept used to describe the knowledge, skills and confidence required by individuals to manage their own health and use medical services (35). Research shows that high levels of patient activation can improve the effectiveness of using digital medical technology, enabling patients to actively explore the functions of digital health services and improve cognitive efficiency (36).

The EuroQol 5-Dimension (EQ-5D) Scale is one of the most effective tools for assessing individual health status (37). Older people with poor health status need more continuous monitoring and long-term health management, so their utilization of digital health services may be higher. Based on this, this study incorporates the three core variables of eHealth literacy, patient activation level, and patient health status into the Technology Acceptance Model.

Put forward the following research hypothesis:

Perceived Usefulness is one of the most core variables in the TAM, which, together with perceived ease of use, determines attitude and behavioral intention (32, 38). Perceived usefulness reflects the user’s assessment of the potential value of a technology in achieving its goals (39). Among older adults, the core of perceived usefulness is that they subjectively believe that digital medical technology can effectively improve health management, improve quality of life, and better cope with health challenges. Research shows that the perceived usefulness in the TAM has a positive impact on attitude (40). Perceived usefulness may also directly affect the adoption of technology. A study shows that there is a significant correlation between the perceived usefulness of auxiliary technology in older adults and their use (r = 0.160; *p* = 0.01).

Therefore, the following assumptions are put forward:

*H1a*: Perceived usefulness directly promotes the use of digital health services by older adults.

*H1b*: Perceived usefulness has a positive impact on the attitude of digital health services by older adults.

Perceived ease of use is the core concept of the TAM, which refers to the degree to which users subjectively believe that a technology requires less effort, is easy to operate, and easy to learn (41). Its core reflects the assessment of the mental effort required by older adults when using digital tools. Within the framework of the TAM, perceived ease of use can not only directly improve the positive attitude of older adults towards such technologies, but also indirectly affect their attitudes and behavioral intentions through perceived usefulness. A relevant study shows that the perceived ease of use of clinical department patients on the online medical system directly affects their attitude towards the tool (*β* = 0.940, *p* < 0.001), and indirectly improves their attitude and behavioral intention through perceived usefulness (*β* = 0.718, *p* < 0.001) (42).

Therefore, the following assumptions are put forward:

*H2a*: Perceived ease of use has a positive impact on older adults’ attitudes toward digital health services.

*H2b*: Perceived ease of use indirectly affects the use of digital health services through its positive impact on perceived usefulness.

In the TAM, attitude is defined as the user’s emotional evaluation or preference for the use of a specific technology (39). It reflects the degree of positive or negative emotions held by users towards technology, and this positive attitude will promote individuals to have a stronger willingness to use it (43). At the same time, the behavioral intention will promote the final adoption behavior. Research shows that in patients with chronic diseases, behavioral intention has a significant positive impact on final awareness (*β* = 0.270, *p* < 0.001), want (*β* = 0.257, *p* < 0.001), and adoption (*β* = 0.302, *p* < 0.001) (44).

Therefore, the following assumptions are put forward:

*H3*: Attitude has a positive impact on behavioral intentions.

*H4*: Behavioral intentions have a positive impact on the older adults’ awareness, want, and adoption of digital health services.

eHealth Literacy was proposed by Norman and Skinner in 2006 and includes six components: traditional literacy, information literacy, media literacy, health literacy, computer literacy, and scientific literacy (45). It is reflected in the ability of individuals to search for, understand, and evaluate health information through electronic media and use this knowledge to solve health problems (34). Individuals with a high level of eHealth Literacy are more confident in finding and understanding health information and in using health platforms, and their acceptance and adoption of technology are therefore likely to be higher. Research shows that eHealth Literacy is one of the strongest predictors of individual technology acceptance, and is significantly associated with behavioral intention (46, 47).

Therefore, the following assumptions are put forward:

*H5a*: eHealth Literacy directly promotes the use of digital health systems by older patients.

*H5b*: eHealth Literacy increases the use of digital health services by older patients by improving behavioral intentions.

The concept of patient activation level was first proposed by Hibbard et al. in 2004 (48). It refers to patients’ believe that they play a key role in health management and have the knowledge, skills, and confidence needed to take initiatives to maintain and improve health. In addition, it includes the patient’s ability to adhere to health management behavior under stress or in an emergency. This concept requires patients to actively participate in their own medical care rather than relying passively on medical service providers (35). Its specific contents include a comprehensive understanding of one’s own disease, confidence in obtaining reliable health information, taking the initiative to maintain a healthy lifestyle, actively participating in treatment decision-making, feedback on changes in the condition to medical professionals, and effectively obtaining medical resources and choosing medical services (49–52). In addition, individuals with low levels of patient activation often find it difficult to implement systematic health information processing strategies in the face of complex digital health information, thus limiting their ability to use eHealth Literacy for independent health management (53).

Therefore, the following assumptions are put forward:

*H6a*: Patient activation directly affects the use of digital health services.

*H6b*: Patient activation improves the use of digital health services among older adults by affecting eHealth Literacy.

The EQ-5D scale is one of the most widely used multi-attribute utility tools for assessing health status and calculating quality-adjusted life years (37). It classifies individual health status through five dimensions: mobility, self-care, usual activities, pain or discomfort, and anxiety or depression. Among older adults, poorer health status is often associated with greater need for medical services and long-term health management (54). In this context, digital health services may provide additional convenience and continuity of care. Another study found a significant association between eHealth Literacy Scale (eHEALS) scores and lifestyle-related risk factors among respondents with poorer health status (55). Repeated contact with health services, physicians’ advice, and support from nursing staff may also improve patients’ familiarity with digital tools, thereby lowering the threshold for Internet-based medical care and enhancing eHealth Literacy and subsequent use of digital health services (56).

Based on this, the following hypothesis is put forward:

*H7a*: Poorer health status is associated with higher utilization of digital health services.

*H7b*: Poorer health status increase the use of digital health services by improving eHealth Literacy.

**Figure 1:**
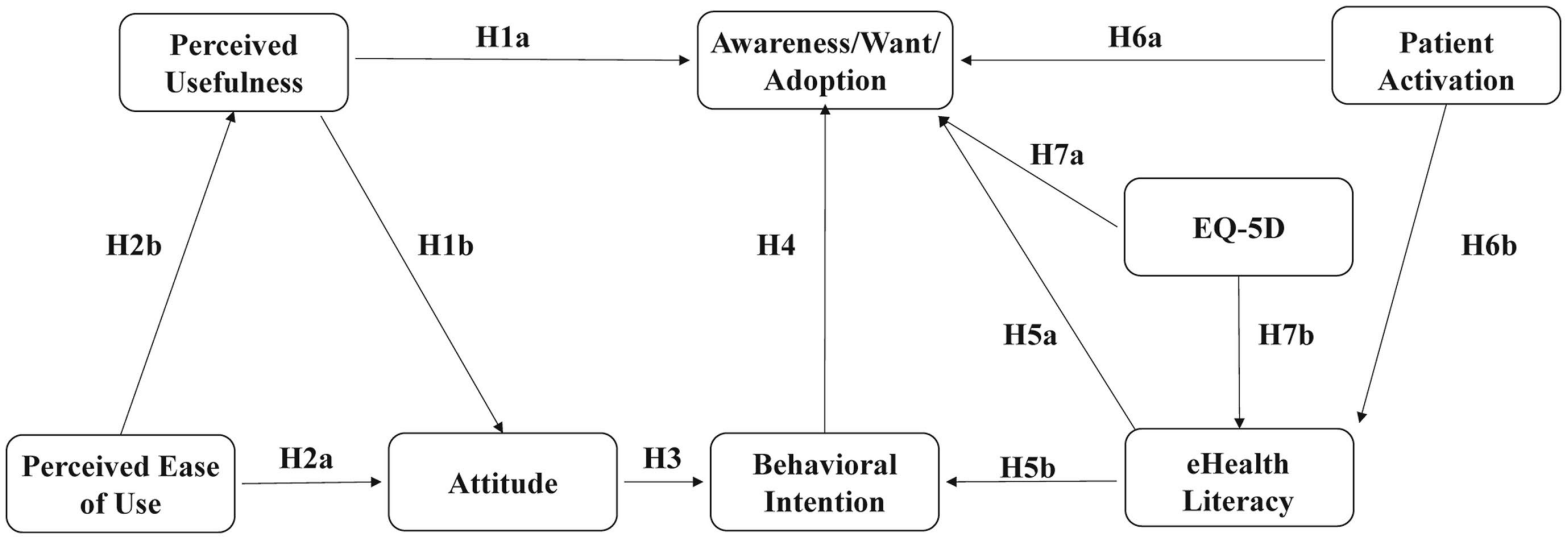
Proposed conceptual model.

This study synthesizes the relationship between the above theoretical perspectives and hypotheses to build a comprehensive conceptual framework (Figure 1).

## 2 Methods

### 2.1 Study population

This cross-sectional study divided digital health services into three functional dimensions: medical information access, convenient medical services, and online health management. In the research design, this study adopted Liang’s adoption model to analyze the use process of older patients, which divided this process into three stages: awareness, want, and adoption. With the help of these frameworks, the spread and influencing factors of digital health use were systematically explored (30, 57).

Based on Lu (58) theoretical constraints on selection bias, this study conducted systematic sampling in three tertiary hospitals in Jinan City, Shandong Province. Aiming to avoid subjective interference, the sampling framework covered all clinical departments, and the double-end interval sampling method was also adopted: the first registered patient was selected on working days, and then one patient was selected from each interval of five patients (skipping the second to fourth patients). This method ensured the objectivity of the sample structure. All participants had signed an informed consent form before being included in the study.

The study subjects were patients aged 60 and above who were conscious and could communicate effectively. Individuals with severe, unstable diseases, cognitive or mental dysfunction, language barriers, or unwillingness to participate in the study were excluded, which would affect their effective participation in the survey to ensure the validity of the data.

The minimum sample size for this cross-sectional survey was determined using the standard approach for estimating a single population proportion. In the absence of a definitive estimate of the utilization rate of digital health services in the study population, a conservative expected proportion of 50% was adopted to yield the maximum sample size. Assuming a 95% confidence level and a margin of error of 5%, the calculation results showed that the minimum sample size to meet the basic research needs was 385 cases. In order to enhance the statistical robustness of the pre-planned subgroup comparison and reduce the loss of effectiveness that may be caused by multiple test corrections, this study extended the recruitment cycle. In the end, the sample size exceeded the minimum requirement, a total of 451 cases. This method aims to improve the ability of research to identify differences in clinical significance in vulnerable people.

### 2.2 Questionnaire design

This study used quantitative survey methods to design questionnaires to comprehensively evaluate the popularity of digital health services, user needs, and actual use of patients. The questionnaire was developed based on previously published literature and established instruments, including the Technology Acceptance Model (33, 39), EQ-5D (37), eHEALS (34), the Patient Activation Measure (PAM) (48), and awareness–want–adoption-gap (AWAG)-based service-stage items (30, 57). A structured Chinese questionnaire was designed based on domestic and foreign standards, aiming to better understand the penetration of digital health services among older population. Collect data from multiple angles through a series of carefully designed questions. See Tables 1, 2 for detailed survey items. Table 1 contains the 5-point Likert-scale items, whereas Table 2 presents the binary service-stage items used for AWAG matrix analysis (14, 57). See Supplementary Table 1 for the complete questionnaire. Before the formal survey, the questionnaire was reviewed by the research team, including public health faculty members, and clinicians. The team discussed whether the items were appropriate for hospitalized older adults and revised the wording, item order, and response options accordingly.

**Table 1:** Measurement items of the 5-point Likert scale form construct.

Construct	Items	Reference
Perceived usefulness (PU)		(33)
PU1	Digital health services are helpful in daily health management.	
PU2	There are significant advantages to using digital health services to manage my health better.	
PU3	Using digital health services is beneficial to me.	
PU4	Using digital health services is of high value to my healthcare.	
Perceived ease of use (PEOU)		(33)
PE1	Learning how to use digital health services is easy for me.	
PE2	My interactions using the Internet to search for health information are straightforward to understand.	
PE3	The Internet is very flexible and easy to use when accessing health information.	
PE4	Overall, digital health services are easy to use.	
Attitude towards Digital Medical Services (Attitude, At)		(33)
At1	Using digital health services is a good idea.	
At2	Using digital health services is a wise and recommended decision.	
At3	I like using digital health services.	
EQ-5D Scale		(22)
EQ-5D1	Action	
EQ-5D2	Take care of yourself.	
EQ-5D3	Daily activities (work, study, household chores)	
EQ-5D4	Pain/discomfort	
EQ-5D5	Anxiety (nervousness, restlessness)/depression	
The eHealth Literacy Scale (eHEALS)		(34)
eH1	I know how to go online to find useful health resource information.	
eH2	I know how to use the Internet to answer my health questions.	
eH3	I know what health resource information is available on the Internet.	
eH4	I know where to get helpful information about health resources on the Internet.	
eH5	I know how to use the information I get about health resources on the Internet to help myself.	
eH6	I have the skills to evaluate good or bad information about health resources on the Internet.	
eH7	I can differentiate between high-quality and low-quality health resource information on the Internet.	
eH8	I am confident in using web-based information to make health-related decisions.	
Behavioral Intention towards Digital Medical Services (BI)		(33)
BI1	I plan to use digital health services in the future.	
BI2	I believe that I will use digital health services in the future.	
BI3	I intend to use digital health services in the future, for example, after I am discharged from the hospital.	
Patient activation measure (PAM)		(48)
PAM1	Generally speaking, I am the person responsible for taking care of my own health.	
PAM2	The fact that I have played a positive role in my own healthcare is the most important thing that affects my health.	
PAM3	I believe I can prevent or reduce problems related to my health.	
PAM4	I know what each of my prescription drugs does.	
PAM5	I feel confident that I can tell if I need to go to the doctor or if I can deal with health issues on my own.	
PAM6	I believe I can tell my doctor my concerns, even if he or she does not ask.	
PAM7	I believe I can adhere to the medical treatment I may need to do at home.	
PAM8	I understand my health problems and their causes.	
PAM9	I know what treatments are available for my health problems.	
PAM10	I have been able to maintain (keep up with) lifestyle changes such as eating right or exercising.	
PAM11	I know how to prevent my health problems.	
PAM12	I believe in figuring out solutions to new problems with my health when they arise.	
PAM13	I believe I can maintain lifestyle changes such as eating right and exercising, even in times of stress.	

**Table 2:** Measurement items of the dichotomous scale form construct.

Construct	Items	Item sources
Awareness/Want/Adoption of digital health services (Aw, Wa, Ad)		(30, 57)
Aw1/Wa1/Ad1	Online collection of disease/health information	
Aw2/Wa2/Ad2	Online collection of doctor/hospital information	
Aw3/Wa3/Ad3	Online collection of doctor-patient evaluation information (patients’ evaluations of doctors)	
Aw4/Wa4/Ad4	Online consultation (graphic and text consultation)	
Aw5/Wa5/Ad5	Online communication with fellow patients about illness in groups or forums	
Aw6/Wa6/Ad6	Online appointment for medical registration	
Aw7/Wa7/Ad7	Online payment of medical expenses	
Aw8/Wa8/Ad8	Online access to electronic medical records (EMRs) and examination reports	
Aw9/Wa9/Ad9	Online appointment for examinations or surgeries	
Aw10/Wa10/Ad10	Online purchase of pharmaceutical products (excluding health supplements)	
Aw11/Wa11/Ad11	Online chronic disease monitoring and management	
Aw12/Wa12/Ad12	Online hospitalization appointment	

### 2.3 Data collection

The questionnaire data were collected through face-to-face surveys conducted in hospital wards by students from the School of Public Health, Shandong University. All investigators received standardized training and mastered the correct procedures for questionnaire administration, data recording, and participant communication. The survey collected general demographic characteristics, including age, gender, marital status, residence, educational duration, and household economic status, as well as health-related and digital health-related information. The main measurement tools included EQ-5D, the Patient Activation Measure, eHEALS, Technology Acceptance Model-related constructs, and awareness–want–adoption stage items for digital health services. The questionnaire was administered in Chinese, and standardized explanations were provided when necessary without guiding participants’ responses. This study was approved by the School of Public Health Ethics Committee, Shandong University (Approval No. LL20230602), and all participants provided written informed consent before participation. After data collection, completed questionnaires were reviewed and checked by team leaders before data entry to ensure consistency and accuracy. All questionnaire data were de-identified before analysis and stored securely by the research team. Access to the dataset was restricted to authorized researchers only, and all participant information was handled in accordance with approved ethical and confidentiality requirements.

### 2.4 Data analysis

Data analysis was conducted using IBM SPSS Statistics 27.0.1. For continuous variables, data are presented as mean ± standard deviation (mean ±SD); categorical variables are expressed as frequencies. For data on a 5-point Likert scale, median and interquartile range (IQR) were employed to minimize the impact of outliers and skewness. Intergroup differences were assessed using non-parametric tests: the Mann–Whitney U test for two samples and the Kruskal–Wallis H test for multiple samples. Where Kruskal–Wallis H test results were significant, further pairwise comparisons were conducted using Bonferroni-adjusted post-hoc tests (adjusted *p* < 0.05) (59). All supplementary analyses as well as data visualization were performed using R (version 4.3.3) software. At the same time, this study used the AWAG matrix (14, 57) to deeply analyze the differences in the digital divide in the field of digital health services from the three dimensions of awareness, want, and adoption gap. The calculation formula using the gap is as follows:


$$g(x)=\frac{\min(\mathrm{Pr}(A(x)),\mathrm{Pr}(W(x)))-\mathrm{Pr}(U(x))}{\min(\mathrm{Pr}(A(x)),\mathrm{Pr}(W(x)))}$$

Where Pr(A(x)) denotes the awareness rate of digital healthcare, Pr(W(x)) denotes the want rate of digital healthcare, and Pr(U(x)) denotes the adoption rate of digital healthcare.

**Figure 2:**
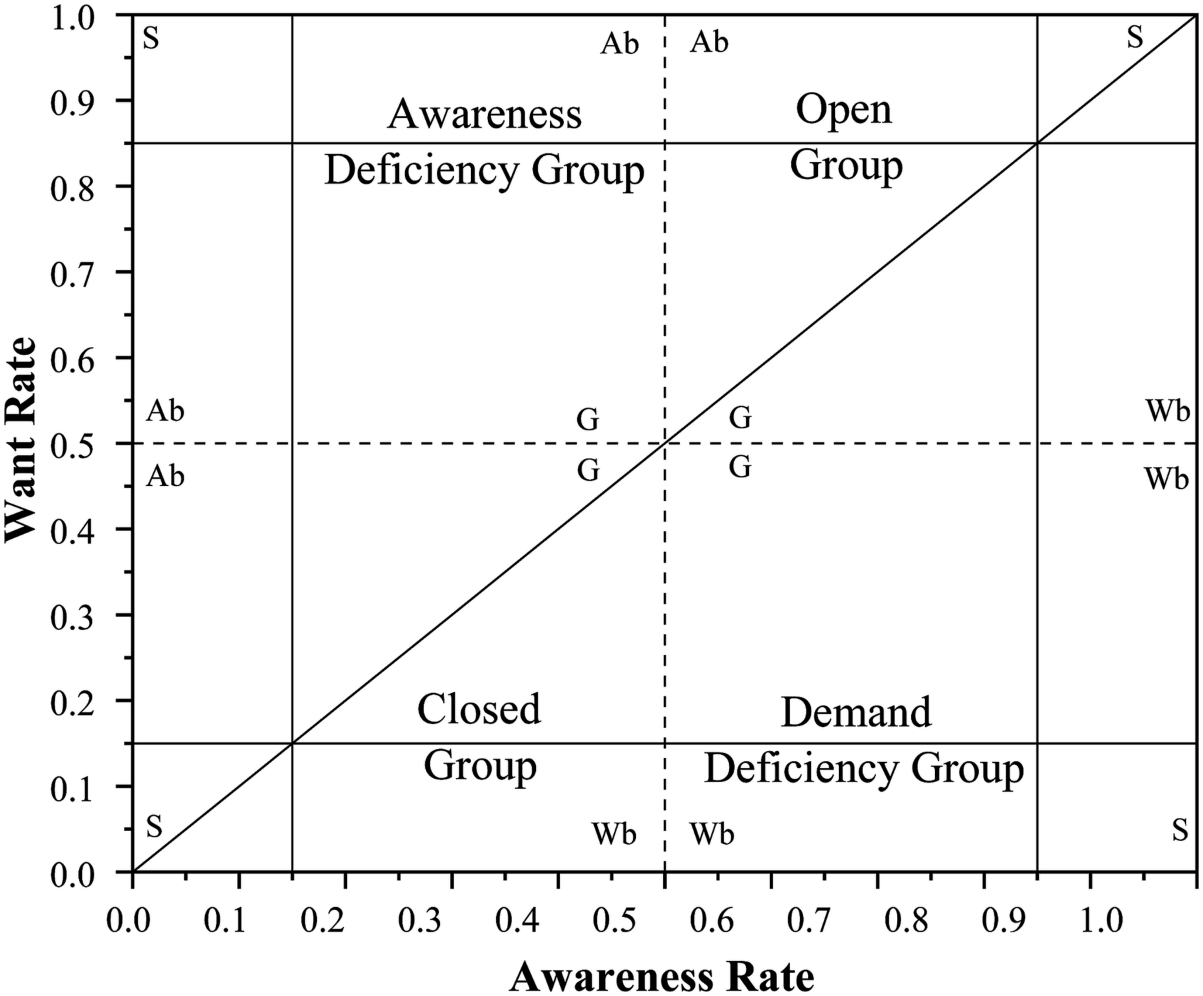
Pattern diagram of the awareness–want–adoption gap (AWAG) segment matrix. Ab, awareness bias; G, generic; S, strong; Wb, want bias.

Based on the Technology Adoption Life Cycle theory (60), the AWAG matrix diagram is segmented according to three thresholds: 15%, 50%, and 85%. The 50% threshold divides awareness and want into high and low segments, respectively, partitioning the entire matrix into four groups: the Open Group (high awareness and want), the Awareness-deficient Group (low awareness), the Want-deficient Group (low want), and the closed group (low awareness and low want). The 15% and 85% groups are further subdivided into four subcategories, as detailed in Figure 2.

In the AWAG matrix diagram, the horizontal axis represents awareness levels, the vertical axis denotes want levels, and the radius of each circle (scaled by a factor of 35 for enhanced visualization) indicates the gap rate. Digital healthcare services are categorized into three functional thresholds: Medical Information Access, Convenient Medical Services, and Online Health Management (Table 3). Origin Pro 2025 software was utilized to generate all of the matrix plots.

**Table 3:** Classification of digital health services.

Categories	Digital health services
Medical information access	Collect disease/health information online.
	Collect doctor/hospital information online.
	Collect medical reviews (patient comments about doctors) online.
	Communicate with patients in groups or forums.
Convenient medical services	Make an appointment online.
	Pay medical fees online.
	View electronic medical records and test reports online.
	Book an appointment for an examination or surgery online.
	Make an appointment for hospitalization online.
	Buy medicines (not healthcare products) online.
Online health management	Consult online (graphical consultation).
	Monitor and manage chronic conditions online.

Structural equation modeling (SEM) was conducted using IBM SPSS AMOS 24.0 to analyze the latent variables (PU, PEOU, attitude, awareness, want, adoption, eHealth literacy, EQ-5D, patient activation). Given the large sample size (*N* = 451), maximum likelihood estimation was employed with Bollen-Stine Bootstrap *p*-value correction (61) (utilizing 2,000 Bootstrap samples) to adjust confirmatory factor analysis (CFA) as well as SEM model fit indices. The corrected model fit proved highly satisfactory (*p* < 0.001). Excellent fit was defined as: chi-square per degree of freedom (χ^2^/df) < 3, Normed Fit Index (NFI) > 0.90, Tucker-Lewis Index (TLI) > 0.90, Adjusted Goodness-of-Fit Index (AGFI) > 0.90, Comparative Fit Index (CFI) > 0.90, Incremental Fit Index (IFI) > 0.90, and Root Mean Square Error of Approximation (RMSEA) < 0.05 (62). Through iterative model refinement based on the data, the corrected overall model successfully achieved a good level of fit.

### 2.5 Validity and reliability

The scales of our research institute have been tested and confirmed to have good reliability. After the data collection was completed, independent professionals use the double coding method to control the data quality, and finally build a verified standardized research database. Except for the Cronbach’s *α* coefficient (>0.7) of the PAM, the Cronbach’s α coefficients of the rest of the scales are higher than 0.9 (between 0.917 and 0.972); the composite reliability (CR) indicators obtained from CFA are also between 0.917 and 0.972. The above quantitative indicators further confirm the robustness of the reliability and validity of the scales. See Table 4 for the detailed analysis results.

**Table 4:** Reflective constructs assessment.

Construct/measure	Unstandard	Factor loading	Cronbach’s alpha coefficient	CR	AVE	MSV	ASV
PU1	1	0.86	0.940	0.9407	0.7989	0.480	0.1604
PU2	1.104	0.901					
PU3	1.139	0.89					
PU4	1.154	0.923					
eH1	1	0.924	0.971	0.9715	0.81	0.794	0.2484
eH2	1.009	0.93					
eH3	0.959	0.892					
eH4	0.959	0.901					
eH5	0.988	0.919					
eH6	0.89	0.883					
eH7	0.858	0.884					
eH8	0.857	0.864					
PE1	1	0.92	0.952	0.9526	0.8341	0.794	0.2413
PE2	0.975	0.915					
PE3	1.029	0.896					
PE4	0.962	0.922					
BI1	1	0.951	0.966	0.9657	0.9038	0.523	0.2207
BI2	0.976	0.943					
BI3	0.986	0.958					
EQ1	1	0.878	0.917	0.9167	0.6914	0.087025	0.0343
EQ2	1.096	0.935					
EQ3	1.081	0.919					
EQ4	0.803	0.74					
EQ5	0.665	0.648					
PAM1	1	0.569	0.747	0.9235	0.4841	0.087	0.0514
PAM2	0.872	0.567					
PAM3	1.226	0.692					
PAM4	1.229	0.589					
PAM5	1.203	0.727					
PAM6	1.021	0.626					
PAM7	1.325	0.754					
PAM8	1.288	0.749					
PAM9	1.414	0.748					
PAM10	1.257	0.723					
PAM11	1.338	0.774					
PAM12	1.442	0.738					
PAM13	1.351	0.739					

PU, perceived usefulness; eH, the eHealth Literacy Scale; PE, perceived ease of use; BI, behavioral intention towards digital medical services; EQ, EQ-5D; PAM, patient activation measure.

## 3 Results

### 3.1 Participant characteristics

**Figure 3:**
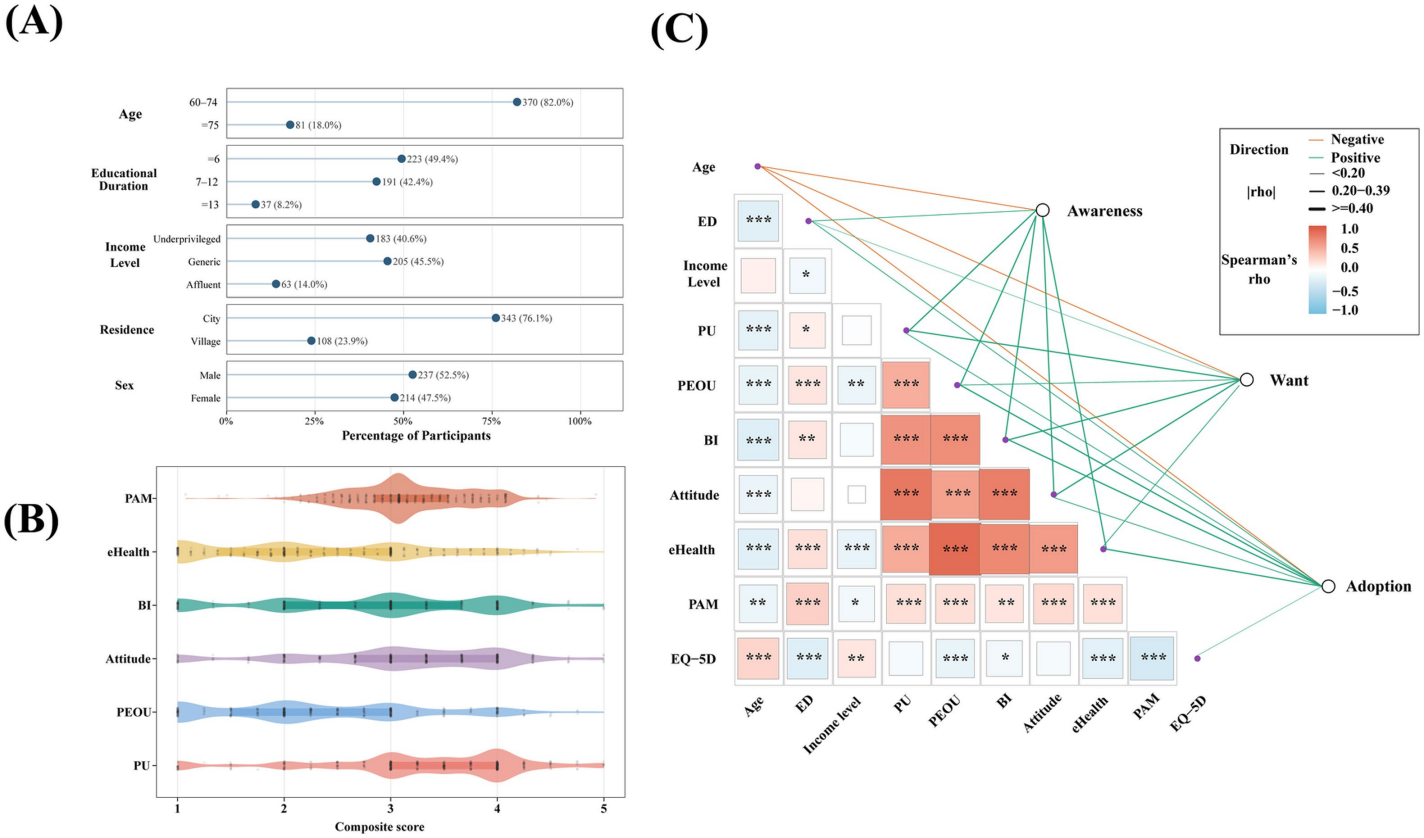
Participant composition, distribution of composite construct scores, and correlation structure of key variables. **(A)** Demographic distribution of the study sample across dimensions such as age, years of education, income level, place of residence and gender (N = 451). **(B)** Distribution of composite scores for Perceived Usefulness (PU), Perceived Ease of Use (PEOU), Attitude, Behavioral Intention (BI), eHealth Literacy, Patient Activation Measure (PAM) and EQ-5D. Higher EQ-5D scores indicate more severe health problems and poorer health status. **(C)** The correlation structure between demographic variables, core constructs and the three-stage outcomes of digital health services (awareness, want and adoption).

This study included a total of 451 older patients, with a fairly balanced gender distribution, where 52.43% were male. The majority of participants (90.00%) were married, and the mean age of the sample was 68.67 years (SD = 7.069). Patients aged between 60 and 74 accounted for 82.10% of the cohort. In terms of education, the average number of years of schooling was 7.49 years (SD = 3.912). It is worth noting that 49.56% of the participants have been educated for 6 years or less, and 42.40% of the participants have been educated for 7–12 years. Figure 3A provides a clearer illustration of the distribution of the sample across dimensions such as age, years of education, income level, place of residence and gender. For more details, please refer to Table 5.

**Table 5:** Sociodemographic characteristics of elder people (*N* = 451).

Variables	Number (%)
Gender	
Male	237 (52.43%)
Female	215 (47.57%)
Age	
60–74	371 (82.10%)
75	81 (17.90%)
Marital status	
Single	2 (0.40%)
Married	406 (90.00%)
Divorced	4 (0.9%)
Widowed	39 (8.6%)
Reside	
City	344 (76.10%)
Village	108 (23.90%)
Educational duration	
6	224 (49.56%)
7 ~ 12	191 (42.40%)
13	37 (8.19%)
Income level	
Very poor or underprivileged	183 (40.4%)
Average level	206 (45.60%)
Above average or relatively affluent	63 (13.9%)
Self-assessment of health status	
Extremely poor or poor	168 (37.2%)
Average level	218 (48.20%)
Good or excellent	66 (14.60%)

In addition to general demographic characteristics, this study further illustrates the distribution patterns of the composite scores for each core construct (Figure 3B). Overall, older patients surveyed rated the potential value of digital health services relatively highly, with perceived usefulness having the highest median score (3.50, IQR = 1.00), followed by patient activation level (3.08, IQR = 0.69), attitude (3.00, IQR = 1.33) and behavioral intention (BI, 3.00, IQR = 1.67). In contrast, the overall levels of perceived ease of use (PEOU, 2.25, IQR = 1.50) and eHealth literacy (2.12, IQR = 1.63) were relatively low.

Meanwhile, the median EQ-5D score was 2.00 (IQR = 1.00). It should be noted that in this study, a higher EQ-5D score indicates more severe health problems and poorer health status; therefore, this result suggests that the overall health burden of the sample is at a relatively low to moderate level. Overall, although older patients generally recognize the potential value of digital health services and possess a certain level of motivation for health management, there remain shortcomings in terms of perceived ease of use and eHealth literacy, which may limit their ability to further translate this subjective recognition into a stable readiness for actual use.

Figure 3C further summarizes the relationship between demographic variables, core constructs and the three-stage outcomes of digital health services (awareness, want and adoption). Overall, awareness was positively correlated with perceived usefulness (*r* = 0.51), behavioral intention (*r* = 0.52), attitude (*r* = 0.46), eHealth literacy (*r* = 0.45) and perceived ease of use (*r* = 0.44), all showing moderate-strength correlations (all *p* < 0.001). Among the three outcomes, “want” showed the strongest correlation with perceived usefulness (*r* = 0.54), whilst also exhibiting significant positive correlations with attitude (*r* = 0.49), behavioral intention (*r* = 0.52), eHealth literacy (*r* = 0.36) and perceived ease of use (*r* = 0.35) (all *p* < 0.001). “Adoption” showed the strongest correlation with behavioral intention (*r* = 0.47), followed by eHealth literacy (*r* = 0.45) and perceived ease of use (*r* = 0.40), whilst correlations with perceived usefulness and attitude were relatively weaker (average *r* ≈ 0.37).

In terms of demographic factors, age was negatively correlated with awareness, want and adoption (*r* = −0.25, −0.19 and −0.22, respectively); years of education were positively correlated with awareness and adoption (both *r* = 0.29), whilst the correlation with want was relatively weaker (*r* = 0.15). Furthermore, eHealth literacy was strongly positively correlated with perceived ease of use (*r* = 0.86) and was significantly positively correlated with behavioral intention, attitude and perceived usefulness (*r* = 0.70, 0.61 and 0.53, respectively).

### 3.2 Matrix analysis and nonparametric tests

**Figure 4:**
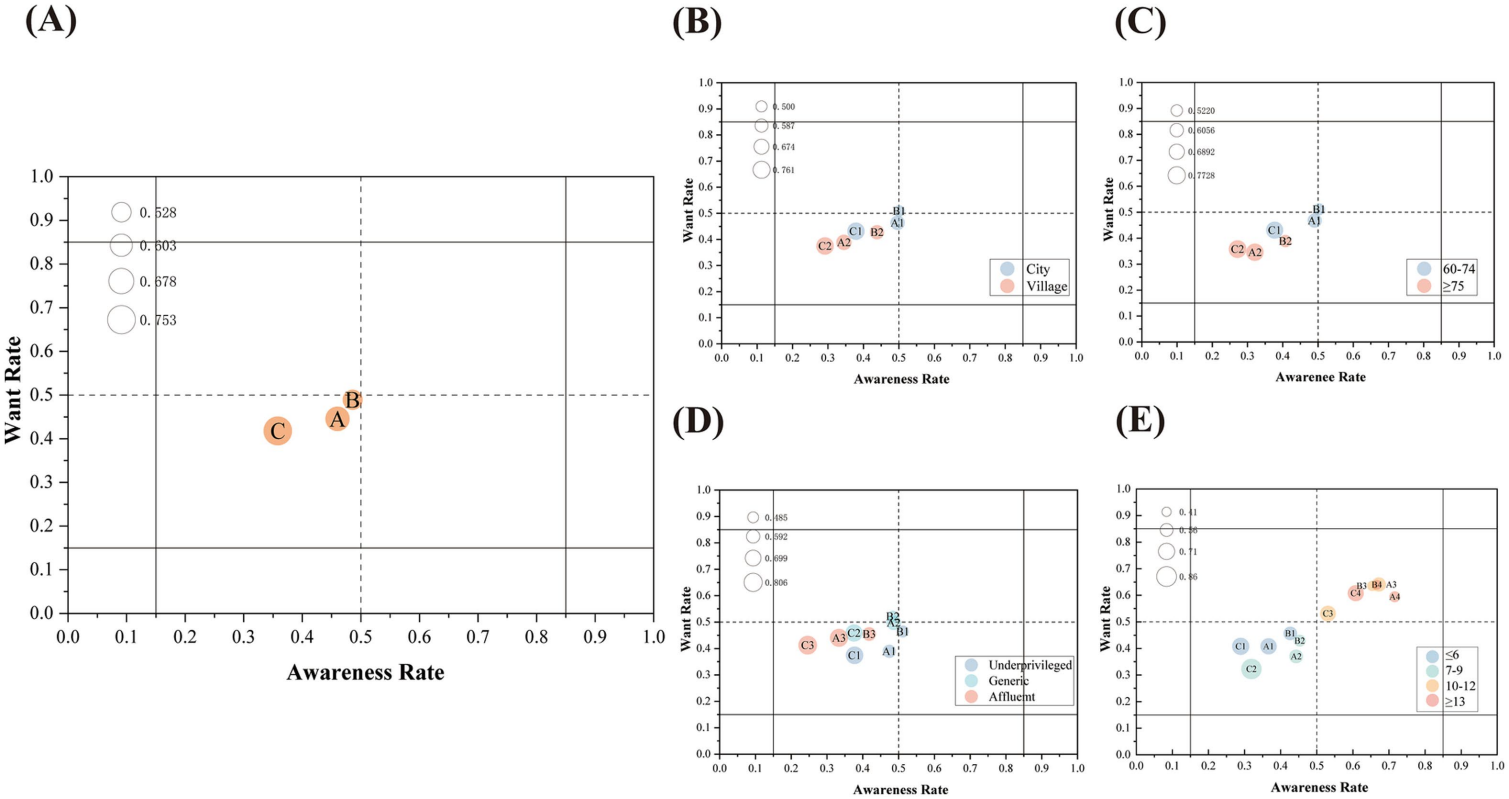
AWAG matrix analysis of digital health services among surveyed patients. **(A)** Overall situation. **(B–E)** Subgroup analyses by the following factors: **(B)** region of residence; **(C)** age; **(D)** income level; and **(E)** years of education. Within each AWAG matrix, the service categories were coded as A, Medical Information Access; B, Convenient Medical Services; and C, Online Health Management. In subgroup panels, numeric suffixes (e.g., A1, A2; B1, B2; C1, C2) indicate the corresponding subgroup-specific positions of each service category.

To ensure the rigor of the analysis, this study stratified the samples according to age, place of residence, income and education level, and verified the applicability of the non-parametric test through the preliminary Kolmogorov–Smirnov analysis, which confirmed that the distribution of all subgroups did not obey the normality (*p* < 0.001). Analysis shows that there is a general use gap among older adults, reaching 59.93%, which is rooted in the disconnect between the relatively high awareness (45.60%) and want (46.27%) and the lower adoption rate (18.27%). The threshold analysis of each functional module shows that convenient medical services perform the best, with the highest awareness (48.60%), want (48.93%) and adoption rate (22.91%), and the lowest gap rate, which is 52.85%. Medical Information Access is secondary. Its awareness (46.01%), want (44.57%) and adoption rate (16.02%) are at a medium level, with a gap rate of 64.05%. In contrast, online health services performed the worst, with the lowest awareness (35.81%), want (41.80%) and adoption rate (8.87%), which eventually led to the highest utilization gap rate, reaching 75.23%. This confirms that in all functional categories, the adoption rate is significantly lower than the awareness and want (Figure 4A).

The analysis based on the place of residence shows that the place of residence of older population is a key factor affecting the utilization of services (Figure 4B). Urban residents are significantly superior to rural residents in many key health indicators. Specifically, in terms of Medical Information Access, the awareness and adoption rate of urban groups are significantly higher (*p* < 0.001, *p* = 0.036, respectively). In the category of convenient medical services, this gap is more significant, and the adoption rate of urban older people is significantly higher (*p* < 0.001). In addition, the awareness of online health management options by the urban older adults is also significantly higher than that of the rural older adults (*p* = 0.035). For details, see Table 6.

**Table 6:** Non-parametric test results by residence.

Residence	City (M, IQR)	Village (M, IQR)	Z	*p*
MIA awareness	0.5 (0.75)	0.25 (0.75)	3.512	<0.001
MIA want	0.5 (1)	0.25 (1)	1.483	0.138
MIA adoption	0 (0.25)	0 (0.25)	2.093	0.036
CMS awareness	0.5 (0.6666)	0.5 (0.7917)	1.572	0.116
CMS want	0.5 (0.8333)	0.3333 (0.8333)	1.832	0.067
CMS adoption	0.1667 (0.5)	0 (0.3333)	3.534	<0.001
OHM awareness	0.5 (0.5)	0 (0.5)	2.112	0.035
OHM want	0.5 (1)	0 (1)	1.226	0.22
OHM adoption	0 (0)	0 (0)	1.387	0.166

M, median; IQR, interquartile range; MIA, medical information access; CMS, convenient medical services; OHM, online health management.

Age-based analysis (Figure 4C) highlights that with the ageing of the older adults group, the service participation declines significantly, and the performance of the 60-74-year-old age group is consistently better than that of the 75-year-old and above. In terms of Medical Information Access, the younger group was significantly higher in terms of awareness, want and adoption rate (*p* < 0.001, *p* = 0.02 *p* = 0.002 respectively). This trend of better performance is also obvious in convenient medical services, and the awareness and adoption rate of the younger group are significantly higher (*p* = 0.03, *p* = 0.045 respectively); in terms of online health management, the 60-74-year-old group also maintains significantly higher awareness (*p* = 0.019). The detailed statistical classification of these age-related differences is shown in Table 7.

**Table 7:** Non-parametric test results by age.

Age	60–74 (M, IQR)	≥75(M, IQR)	Z	*p*
MIA awareness	0.5 (0.75)	0 (0.75)	3.577	<0.001
MIA want	0.5 (1)	0 (0.75)	2.333	0.02
MIA adoption	0 (0.25)	0 (0)	3.165	0.002
CMS awareness	0.5 (0.6666)	0.3333 (0.6667)	2.173	0.03
CMS want	0.5 (1)	0.3333 (0.75)	2.53	0.11
CMS adoption	0.1667 (0.5)	0 (0.3333)	2.006	0.045
OHM awareness	0.5 (0.5)	0 (0.5)	2.336	0.019
OHM want	0.5 (1)	0 (0.75)	1.303	0.193
OHM adoption	0 (0)	0 (0)	1.222	0.222

M, median; IQR, interquartile range; MIA, medical information access; CMS, convenient medical services; OHM, online health management.

When the sample was divided into affluent groups, generic groups and underprivileged groups according to income, the Kruskal–Wallis H test identified significant differences in Medical Information Access (awareness/use rate) and online health management awareness (*p* < 0.05). However, the after-the-fact analysis (Table 8) reveals a counter-intuitive trend: in these categories, the awareness and adoption rate of the affluent group is significantly lower than that of the underprivileged group and the generic group (*p* < 0.05). The matrix analysis in Figure 4D further reinforces this difference, which shows that the usage pattern of affluent individuals is functionally closer to the “closed group.”

**Table 8:** Non-parametric test results by income.

Income	Underprivileged (M, IQR)	Generic (M, IQR)	Affluent(M, IQR)	H	*p*
MIA awareness	0.5 (0.75)	0.5 (0.75)	0.25 (0.75)^*,#^	7.867	0.02
MIA want	0.25 (0.75)	0.5 (1)	0.25 (1)	5.408	0.067
MIA adoption	0 (0.25)	0 (0.25)	0 (0)^*,#^	7.086	0.029
CMS awareness	0.5 (0.6666)	0.5 (0.6666)	0.3333 (0.6667)	3.422	0.181
CMS want	0.5 (0.8333)	0.5 (1)	0.5 (0.8333)	2.672	0.263
CMS adoption	0.1667 (0.5)	0.1667 (0.5)	0 (0.3333)	4.477	0.107
OHM awareness	0.5 (0.5)	0.5 (0.5)	0 (0.5)^*,#^	6.413	0.041
OHM want	0 (1)	0.5 (1)	0.5 (1)	3.565	0.168
OHM adoption	0 (0)	0 (0)	0 (0)	3.137	0.208

M, median; IQR, interquartile range; MIA, medical information access; CMS, convenient medical services; OHM, online health management.

^*^denotes a comparison with the underprivileged income subgroup, where Adj. *p* < 0.05.

^#^represents a comparison with the generic income subgroup, where Adj. *p* < 0.05.

Years of education seems to be a profound driver affecting the cognitive ability of digital health services. Kruskal–Wallis H test and matrix analysis (Figure 4E) confirm that there are significant differences (*p* < 0.05) in all functional thresholds, including awareness, want and adoption. For details, see Table 9. Post-hoc analysis clarifies that seniors with at least 10 years of education (subgroups 10–12 and ≥13) significantly surpassed those with ≤ 9 years (subgroups ≤ 6 and 7–9) across every measured dimension. Concurrently, the 7–9 years subgroup displayed significantly higher awareness in Medical Information Access and Online Health Management than the ≤ 6 years group (*p* < 0.05). Detailed results are presented in Table 9.

**Table 9:** Non-parametric test results by education duration.

Education duration	≤6 (M, IQR)	7—9 (M, IQR)	10—12 (M, IQR)	≥13 (M, IQR)	H	*p*
MIA awareness	0.25 (0.75)	0.5 (0.75)*	0.75 (0.5)^#^	0.75 (0.5)^#^	48.337	<0.001
MIA want	0.25 (1)	0 (0.75)	0.875 (0.75)^#^	1 (1)^#^	24.272	<0.001
MIA adoption	0 (0.25)	0 (0.25)	0 (0.5)^#^	0.25 (0.75)^#^	21.695	<0.001
CMS awareness	0.3333 (0.6667)	0.5 (0.6667)	0.6667 (0.3333)^#^	0.6667 (0.3333)^#^	34.269	<0.001
CMS want	0.5 (0.8333)	0.3333 (0.8333)	0.6667 (0.6667)^#^	0.8333 (0.8333)^#^	19.1	<0.001
CMS adoption	0 (0.3333)	0 (0.3333)	0.3333 (0.3333)^#^	0.3333 (0.5)^#^	36.102	<0.001
OHM awareness	0 (0.5)	0 (0.5)*	0.5 (1)^#^	0.5 (0.75)^#^	35.126	<0.001
OHM want	0.5 (1)	0 (0.5)	0.5 (1)^#^	0.5 (1)^#^	17.295	<0.001
OHM adoption	0 (0)	0 (0)	0 (0.5)^#^	0 (0.5)^#^	30.105	<0.001

M, median; IQR, interquartile range; MIA, medical information access; CMS, convenient medical services; OHM, online health management.

*denotes a comparison between subgroups with an education duration of ≤ 6 years, with Adj. *p* < 0.05.

^#^denotes a comparison between subgroups with an education duration of ≤ 9 years, with Adj. *p* < 0.05.

### 3.3 Normality test

Before performing CFA and SEM estimations, this study first assessed the normality of univariate distributions for each observed item to evaluate the appropriateness of subsequent parameter estimation methods (see Supplementary Table 2). According to Kline, data can be considered approximately normal for SEM analysis when the absolute values of skewness and kurtosis do not exceed 3 and 8, respectively (63). In general, the skewness and kurtosis of most items fell within acceptable ranges; however, dichotomous items such as Awareness and Want exhibited some flattened kurtosis (often negative), though their absolute values remained below the empirical thresholds. Notably, certain items in Adoption displayed more pronounced positive skewness and peaked kurtosis: Ad5 (skewness = 3.227, kurtosis = 8.453), Ad9 (skewness = 3.491, kurtosis = 10.231), Ad11 (skewness = 3.421, kurtosis = 9.743), and Ad12 (skewness = 3.972, kurtosis = 15.276), all of which exceeded the thresholds. Given that these deviations only exist in a few projects and reflect the actual distribution characteristics of the digital health services behavior of older population, this study retains the above items to ensure the full coverage and measurement validity of the conceptual content, so as to maintain the validity of the model. In addition, after excluding these items, the errors generated by the SEM are negligible (Δχ^2^/df = 0.074; ΔRMSEA = 0.001).

### 3.4 Reliability, validity, and confirmatory factor analysis

**Figure 5:**
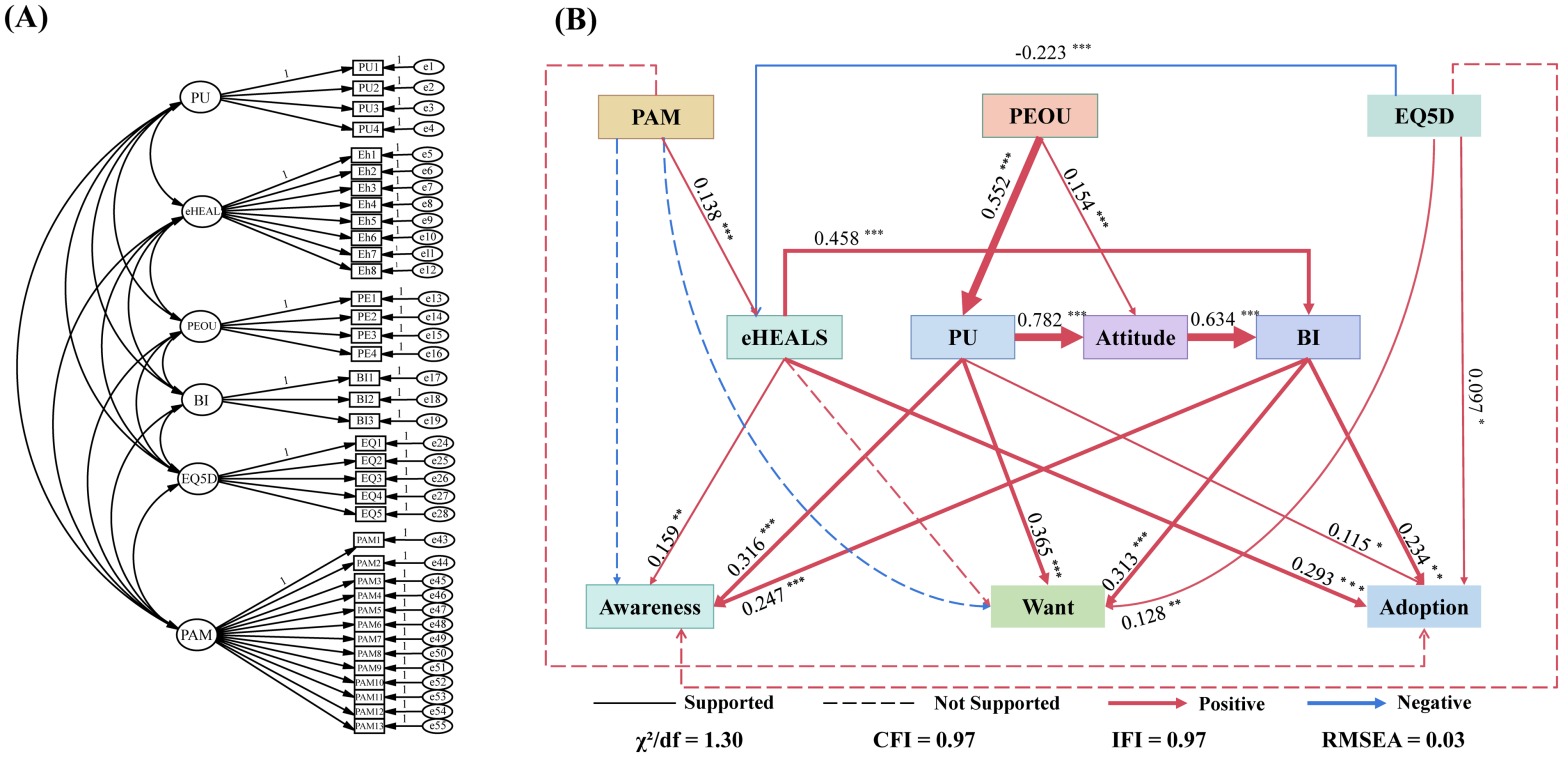
Measurement and structural model analysis. **(A)** Confirmatory factor analysis (CFA) of the measurement model, showing the latent constructs and their observed indicators included in the SEM. **(B)** Final structural equation model showing the hypothesized relationships among the study constructs. Values on the paths are standardized regression coefficients. Solid lines indicate statistically supported paths (*p* < 0.05), whereas dashed lines indicate unsupported paths (*p* ≥ 0.05). Red lines denote positive associations, blue lines denote negative associations, and line width is proportional to the absolute magnitude of the standardized regression coefficient (|*β*|). Higher EQ-5D scores indicate poorer health status.

This study employed CFA to examine the measurement model (see Figure 5A), assessing the measurement validity of each observed variable for latent variables. The overall model fit achieved an excellent level: χ^2^ = 3208.82, df = 2462.00, χ^2^/df = 1.30, RMSEA = 0.03, CFI = 0.97, TLI = 0.97, IFI = 0.97; whilst the NFI was relatively low at 0.90. Considering the model’s complex structure and substantial degrees of freedom, the overall fit is deemed excellent and suitable for subsequent analysis.

The standardized factor loadings for each item were generally high, with most exceeding 0.70 (64). Relatively lower loadings were observed primarily in certain PAM items (PAM1 = 0.569, PAM2 = 0.567, PAM4 = 0.589) and EQ-5D’s EQ5 (0.648). However, all remained above the minimum acceptable threshold of 0.50 and were retained to preserve construct content coverage and measurement validity. Further reliability and validity results are presented in Table 4. Overall internal consistency across constructs was generally good (for example, high Cronbach’s *α* for PU, eHealth literacy, PEOU, BI, and EQ-5D; PAM at 0.747), with CR generally at a high level (64, 65); except for PAM, the average variance extracted (AVE) values for all constructs exceeded 0.50 (PU = 0.799, eHealth literacy = 0.810, PEOU = 0.834, BI = 0.904, EQ-5D = 0.691). Although PAM’s AVE was slightly lower (0.484), its CR was high (0.924), overall supporting its convergent validity (64). Regarding discriminant validity, both maximum shared variance (MSV) and average shared variance (ASV) were lower than AVE, and each construct’s square root of AVE (0.696–0.951) exceeded its correlation coefficients with other constructs (Table 10). Although eHealth literacy showed a high correlation with PEOU (*r* = 0.891), it did not exceed their respective square root of AVE values (0.900, 0.913), indicating statistical distinguishability between the two constructs (65). In summary, the reliability and validity of the measurement model are generally acceptable, providing a reliable foundation for subsequent SEM analysis.

**Table 10:** Descriptive statistics and intercorrelations of the constructs.

Construct	PU	eHealth	PEOU	BI	EQ	PAM
PU	**0.799**					
eHealth	0.558	**0.81**				
PEOU	0.553	0.891	**0.834**			
BI	0.693	0.723	0.702	**0.904**		
EQ	−0.026	−0.259	−0.215	−0.109	**0.691**	
PAM	0.132	0.199	0.221	0.164	−0.295	**0.484**
The Square Root of AVE	0.894	0.900	0.913	0.951	0.831	0.696

PU, perceived usefulness; eH, the eHealth Literacy Scale; PE, perceived ease of use; BI, behavioral intention towards digital medical services; EQ, EQ-5D; PAM, patient activation measure; AVE, average variance extracted. Bold values indicate the average variance extracted (AVE) for each construct.

### 3.5 Structural equation model

The structural equation model was estimated to test the hypothesized relationships. Overall, the model demonstrated acceptable fit to the data (χ^2^/df = 1.30, RMSEA = 0.03, CFI = 0.97, IFI = 0.97, TLI = 0.97, NFI = 0.90, Goodness-of-Fit Index (GFI) = 0.90, AGFI = 0.89). Based on the significance judgement hypothesis of the standardized path coefficient (*β*) whether the hypothesis is valid, the path of *p* > 0.05 is considered unsupported (Table 11). The results show that perceived usefulness has a significant positive impact on the three stages of the use of digital health services: awareness stage *β* = 0.316 (*p* < 0.001), want stage *β* = 0.365 (*p* < 0.001), and adoption stage *β* = 0.115 (*p* = 0.026); therefore, H1a is supported. At the same time, the influence of perceived usefulness on attitude is stronger (*β* = 0.781, *p* < 0.001), which supports H1b. Perceived ease of use has a direct positive impact on attitude (*β* = 0.154, *p* < 0.001), and significantly improves perceived usefulness (*β* = 0.552, *p* < 0.001), supporting H2a and H2b. This shows that perceived ease of use is further associated with the use of digital health services by improving perceived usefulness. Further analysis found that attitude significantly predicts behavioral intention (*β* = 0.634, *p* < 0.001) and supports H3; behavioral intention then promotes awareness (*β* = 0.247, *p* < 0.001), want (*β* = 0.313, *p* < 0.001), and adoption (*β* = 0.234, *p* = 0.001), so it supports H4.

**Table 11:** Hypothesis testing results of the research mode.

Hypothesis	Pathways			Coef β#	S.E.	C.R.	*p*	Decision
H1a	Awareness	<---	PU	0.316	0.021	5.521	<0.001	Supported
	Want	<---	PU	0.365	0.024	6.987	<0.001	Supported
	Adoption	<---	PU	0.115	0.017	2.234	0.03	Supported
H1b	Attitude	<---	PU	0.781	0.038	19.126	<0.001	Supported
H2a	Attitude	<---	PEOU	0.154	0.03	4.519	<0.001	Supported
H2b	PU	<---	PEOU	0.552	0.044	12.082	<0.001	Supported
H3	BI	<---	Attitude	0.634	0.036	17.675	<0.001	Supported
H4	Awareness	<---	BI	0.247	0.025	3.552	<0.001	Supported
	Want	<---	BI	0.313	0.029	4.963	<0.001	Supported
	Adoption	<---	BI	0.234	0.021	3.192	0.001	Supported
H5a	Awareness	<---	eHEALS	0.159	0.018	2.801	0.005	Supported
	Want	<---	eHEALS	−0.024	0.021	−0.683	0.495	Not Supported
	Adoption	<---	eHEALS	0.293	0.016	4.711	<0.001	Supported
H5b	BI	<---	eHEALS	0.458	0.03	13.33	<0.001	Supported
H6a	Awareness	<---	PAM	−0.086	0.037	−1.395	0.16	Not Supported
	Want	<---	PAM	−0.073	0.042	−1.156	0.25	Not Supported
	Adoption	<---	PAM	0.003	0.031	1.044	0.30	Not Supported
H6b	eHEALS	<---	PAM	0.138	0.120	2.812	0.005	Supported
H7a	Awareness	<---	EQ-5D	0.033	0.018	0.619	0.536	Not Supported
	Want	<---	EQ-5D	0.128	0.021	3.044	0.002	Supported
	Adoption	<---	EQ-5D	0.097	0.016	2.168	0.03	Supported
H7b	eHEALS	<---	EQ5D	−0.223	0.059	−5.369	<0.001	Supported

^#^Standardized path coefficients; S.E., standard error; C.R., critical ratio.

Regarding the variables related to individual ability, eHealth literacy has a significant positive impact on awareness (*β* = 0.159, *p* = 0.005) and adoption (*β* = 0.293, *p* < 0.001), but has no significant impact on want (*β* = −0.024, *p* = 0.495), so it partially supports H5a. At the same time, eHealth literacy significantly affects behavioral intentions (*β* = 0.458, *p* < 0.001) and supports H5b. In contrast, the direct impact of patient activation level on awareness (*β* = −0.086, *p* = 0.163), want (*β* = −0.073, *p* = 0.248), and adoption (*β* = 0.003, *p* = 0.297) is not significant, and H6a is not supported. However, the patient’s activation level has a significant positive impact on eHealth literacy (*β* = 0.138, *p* = 0.005), supporting H6b. This shows that the association between the patient’s activation level and the use of digital health services is mainly realized through the path of eHealth literacy and follow-up intention (Table 11; Figure 5B).

## 4 Discussion

### 4.1 Key findings

By expanding the TAM, this study has built a new framework for understanding the adoption of digital health services for older adults. We add eHealth literacy as the baseline of competence on the basis of the classic model, while incorporating the patient’s activation and health status to capture the subtle differences in motivation, trust, and demand intensity. The key is that the framework divides the adoption life cycle into three stages of “awareness-want-adoption” and classifies services according to functional dimensions (medical information acquisition, convenient medical services, online health management). This detailed division provides a structural basis for accurately identifying where and why older adults withdraw from the process of receiving digital health services.

Using this two-dimensional perspective, this study finds different usage patterns and rejection phenomena. Most notably, we have observed a significant utilization gap, that is, the awareness and want of older population for services are much higher than their actual adoption rate. This disconnection of “awareness-want-adoption” reflects the existence of significant structural obstacles and skills-related obstacles, which are consistent with the digital divide. At the same time, it also highlights the need to improve the availability of digital health services and carry out targeted digital training. In addition, the analysis also found an unexpected difference: the adoption rate of wealthy people in medical information access and online health management is extremely low. These insights provide policymakers with a detailed empirical basis that interventions must address specific dysfunctions and socio-economic paradoxes rather than view the older population as a whole.

In addition to the functional dimension analysis of the awareness, want, and adoption of digital health services based on matrix analysis, this study further introduces the structural equation model method to model and test the theoretical relationship. By combining matrix analysis with SEM, this method reveals the deep reality hidden in the data. First of all, matrix analysis points out the specific differences between subgroups. For example, the gap in the use of online health management is the most serious (75.23%). Secondly, the SEM confirms that eHealth literacy directly promotes adoption, not just affects demand. Third, the combination of the two methods clarifies the specific obstacles and difficulties faced by older adults in using digital health services. The combined use of stage-based AWAG analysis and SEM allowed us not only to identify where older adults disengaged across awareness, want, and adoption, but also to examine the potential mechanisms underlying these discontinuities.

At the same time, the results of this study also present some highlights worthy of attention. Matrix analysis shows that older adults who have been educated for more than 9 years are significantly superior to the older adults who have been educated for ≤9 years in all dimensions of using digital health services, which shows that education is the keyway to achieve digital health equity (66). Unlike previous studies, the utilization rate of convenient medical services among older adults is the highest, surpassing access to medical information. This may be because convenient medical services are more in line with the actual medical needs of older adults, have fewer operating obstacles, and can solve practical problems more directly (such as appointment registration, queuing, and physical consumption). In contrast, access to medical information requires a higher level of information technology and cognitive processing ability. Due to the limited digital literacy of older adults and the lack of user-friendliness of the interface, its utilization rate is relatively low (67). The lowest utilization rate of online health management may be because it is difficult for patients to make long-term behavioral changes, and there is an inherent sense of distrust (68). Regarding the counterintuitive phenomenon that the utilization rate of digital health services of the wealthy older adults is lower than that of the poor group, it can be explained as follows: the wealthy older population may be more inclined to high-quality offline medical services (such as specialized outpatient clinics and private medical care), resulting in a decrease in dependence on digital health services, thus reducing the utilization rate of digital health services (13). In addition, wealthy older people may pay more attention to privacy issues, have a stronger tendency to avoid risk, and are more sensitive to digital health data security, privacy leakage, and personal information abuse. This difference in digital trust and perceived risk may prevent it from adopting digital health services (67, 69).

To improve the utilization rate of digital health services for older population, digital skills training is particularly important, taking the obstacles they face in the process of “awareness-want-adoption” into account. Previous studies have shown that training programs to improve the ability of older adults to use computers and the Internet can help bridge the digital divide (12, 70), thereby enhancing their confidence in using such technologies. To narrow the gap between these three stages, it is also necessary to improve the practicality and user-friendliness of digital health services functions. Especially in terms of online health management, the design of digital health services interfaces should be more in line with the needs of older adults, such as simplifying the operation process, designing intuitive health monitoring tools (such as automatic recording and reminder functions), and providing voice or graphic guidance to reduce barriers to use (71). At the same time, it is necessary to strengthen platform data security and privacy protection measures, which is beneficial to address the concerns of wealthy older people about the security and privacy leakage of digital health data. Strengthening data security and transparent information protection policies can effectively build the trust of older adults in digital health services, thereby improving their willingness to use them (72). In addition, considering the preference of wealthy older people for high-quality offline medical services, improving the medical quality of digital health services is also a key way to promote their adoption (72).

In SEM, we observed that PAM did not exert a direct effect on the adoption of internet-based healthcare services. This aligns with prior evidence: within primary care samples, PAM may increase interest in telemedicine services but does not significantly influence actual use (*p* = 0.671) (73). There was no difference in PAM levels between users and non-users of digital health services systems, and prospective studies also failed to identify a stable association between patient activation and sustained usage (74, 75). However, a nationally representative survey in the United States revealed that higher levels of patient activation were associated with greater likelihood of accessing multiple types of online health information (including electronic health records, quality comparison tools, disease and prevention information, etc.) within the past 12 months (76). We propose that eHealth literacy may mediate the relationship between higher patient activation and the ultimate adoption of digital health services. Research by Zhu et al. (77) indicates that patient activation directly influences the seeking of online health information (*β* = 0.39, *p* < 0.05). Older patients with higher activation levels tend to place greater emphasis on their role and status in disease management and are more willing to proactively seek health information with purpose (77). They tend to actively search for health information online, strictly assess its credibility, and apply it effectively to health management, significantly improving the utilization rate of digital health services. On the contrary, individuals with low patient activation often lack systematic health information processing strategies and find it difficult to effectively encode, store, and retrieve complex digital health information (53). In addition, considering that the sensory and cognitive functions of older patients may have varying degrees of decline, these changes directly affect their ability to learn, understand, and apply digital health technologies. With the aim of reducing this impact on eHealth literacy, we suggest that hospitals develop digital health interfaces suitable for older adults (such as adjusting the font size and simplifying the operation process). At the same time, incentive interventions should be implemented to improve the activation level of patients. This can be achieved through health education, health guidance, or motivational interviews to enhance the confidence and active participation of older patients in self-health management (78).

Interestingly, in the SEM, improving the eHealth literacy of the older significantly increases their awareness and actual adoption of digital health services, but it does not directly translate into a stronger willingness to use it. Previous studies have shown that individuals with high eHealth literacy usually have stronger information retrieval and technical operation capabilities. This enables them to discover and understand online medical services through digital channels, so as to raise awareness of such services. At the same time, it also helps them overcome technical obstacles such as device installation, application configuration, and data interpretation, and promotes smoother adoption. However, the use of willingness involves more complex emotional and psychological factors, such as personal values, emotional experiences, cultural cognition, and personality traits. These factors may weaken the impact of eHealth literacy on the will. Compared with young people, older adults tend to experience psychological pressure and emotional costs when using new technologies, such as fear of making mistakes, operational setbacks, or lack of interpersonal interaction. These negative emotions do not disappear automatically with the improvement of knowledge or skills. Without psychological support and guidance for older adults, such as encouragement and patient guidance during use, even individuals with a high literacy level may be hesitant to adopt digital health services (79).

### 4.2 Limitations

Several considerations should be taken into account when interpreting the findings of this study. First, the sample was drawn from hospitalized older adults with chronic diseases in tertiary hospitals in Jinan. This sampling frame is well suited to the present research question, but it also means that the findings primarily reflect digital health service use in hospital-based care contexts and should be generalized to other settings, such as primary care, community-based management, or regions with different healthcare infrastructures, with caution. Second, the cross-sectional design identifies stage-specific associations among awareness, want, and adoption, but it does not capture how individuals move across these stages over time. Accordingly, the temporal ordering and stability of these relationships require further confirmation in longitudinal settings. Third, although the questionnaire was refined through repeated review by supervisors, public health faculty members, and clinicians to improve its relevance and clarity for hospitalized older adults, further validation in independent samples and broader service contexts would strengthen the robustness of the measurement framework. In addition, because the study relied mainly on self-reported information collected in real-world ward settings, the findings remain subject to reporting bias and to the practical constraints inherent in field-based recruitment and implementation. The SEM approach used here effectively clarified major structural relationships, but more complex contextual influences—such as service quality, privacy protection, and institutional support—merit deeper examination in future work.

Future research would benefit from multi-region and multi-setting designs, especially studies that include community and primary-care populations alongside hospital-based samples. Longitudinal follow-up, integration of objective behavioral indicators such as platform-use records, and more refined analytical strategies would further strengthen understanding of how older adults progress from awareness to willingness and from willingness to actual adoption of digital health services.

## 5 Conclusion

By expanding the TAM, this study has built a comprehensive digital health adoption framework for older population, incorporating eHealth literacy and patient activation level as the basic driving factor of the path of “awareness-want-adoption.”

Despite the relatively high participation of “convenient medical services,” there is still a significant gap in the use of “online health management,” which especially hinders the adoption of older adults, low-educated, and rural people. It is worth noting that the study has found a counterintuitive trend: the adoption rate of wealthy older adults people is lower, which may be driven by higher privacy concerns and a preference for high-quality offline medical services. In addition, structural analysis shows that although eHealth literacy provides technical capabilities for adoption, it cannot be automatically transformed into the willingness to use it due to persistent psychological disorders and operational anxiety.

In order to bridge the critical “willingness-adoption” gap, interventions must go beyond basic skills training, prioritizing interface design suitable for older adults, psychological support to reduce the fear of use, and strong data security measures to rebuild trust. Our two-dimensional framework (combining functional dimensions and phased analysis) provides a precise roadmap for promoting digital inclusion and maximizing the health and equity of older adults.

## Data Availability

The datasets generated and/or analyzed during the current study are available from the corresponding author on reasonable request.

## Glossary

TAM: Technology acceptance model
TRA: Theory of reasoned action
PU: Perceived usefulness
PEOU: Perceived ease of use
BI: Behavioral intention
AU: Actual use
PAM: Patient activation measure
eHEALS: eHealth Literacy Scale
EQ-5D: EuroQol 5-Dimension Scale
AWAG: Awareness–Want–Adoption Gap
SD: Standard deviation
IQR: Interquartile range
CFA: Confirmatory factor analysis
SEM: Structural equation modeling
CR: Composite reliability
AVE: Average variance extracted
MSV: Maximum shared variance
ASV: Average shared variance
NFI: Normed fit index
GFI: Goodness-of-fit index
AGFI: Adjusted goodness-of-fit index
CFI: Comparative fit index
IFI: Incremental fit index
TLI: Tucker–Lewis Index
RMSEA: Root mean square error of approximation
